# Self-lubricating Al-WS_2_ composites for efficient and greener tribological parts

**DOI:** 10.1038/s41598-017-15297-6

**Published:** 2017-11-07

**Authors:** Vlad Bogdan Niste, Monica Ratoi, Hiroyoshi Tanaka, Fang Xu, Yanqiu Zhu, Joichi Sugimura

**Affiliations:** 1 Faculty of Engineering and Environment, University of Southampton Highfield Campus, Southampton, SO17 1BJ United Kingdom; 2 International Institute for Carbon-Neutral Energy Research, Kyushu University 744 Motooka, Nishi-ku, Fukuoka, 819-0395 Japan; 3 Research Centre for Hydrogen Industrial Use and Storage, Kyushu University 744 Motooka, Nishi-ku, Fukuoka, 819-0395 Japan; 4 College of Engineering, Mathematics and Physical Sciences, University of Exeter, Exeter, EX4 4QF United Kingdom; 5 Faculty of Engineering, The University of Nottingham, Nottingham, NG7 2RD United Kingdom

## Abstract

Due to their mechanical and physical properties, aluminium alloys possess wide potential in the automotive industry, particularly in hot reciprocating applications such as pistons for diesel and petrol engines. WS_2_ particle-reinforced composites could bring further improvements by reducing friction and wear between moving parts. Reducing friction improves efficiency by lowering energy/fuel use, ultimately leading to lower greenhouse gas emissions, while antiwear properties can prolong component life. This study compares for the first time the tribological performance of powder metallurgy-consolidated Al composites reinforced with either IF- or 2H-WS_2_ particles, so as to elucidate their mechanism of action in test conditions similar to those encountered in engine applications. The composites were tested in lubricated reciprocating contacts against AISI52100 steel balls and the impact of WS_2_ could be seen at both 25 and 100 °C. The reduced friction and wear at ambient temperature is due to the predominantly physical mechanism of action of WS_2_, while the best antiwear performance is measured at elevated (standard operating engine) temperatures that promote the chemical reaction of WS_2_ with the aluminium matrix. The investigation focused on studying the wear tracks/scars and the tribofilms generated on the composite and ball with optical profilometry, SEM, XPS and Auger spectroscopy.

## Introduction

Aluminium metal matrix composites (Al MMCs) have recently gathered considerable interest in the search for light-weight materials that can offer better fuel economy, reduced vehicle emissions and increased safety. The excellent mechanical and tribological properties of Al MMCs have led to the development of new components used in aerospace, automotive and marine applications^1–4^.

Al MMCs are composed of an Al alloy matrix and different material particle additions that modify the properties of the composite. Depending on the type of particle constituents, improvements in hardness, tensile strength, wettability, friction properties, wear resistance or load carrying capacity can be achieved^4–6^. The extent of the improvements was found to depend on an application-specific optimal particle size^4,7^, a uniform particle distribution in the Al matrix^4,5,8,9^ and an adequate concentration^3,4,7,10–13^.

Due to the inferior tribological properties of pure aluminium, ceramic particle additions such as SiC and Al_2_O_3_ have been added to Al MMCs to increase the mechanical properties of the Al matrix^3,4,8,9,13,14^.

The downside of these hard additions is a high COF and their abrasiveness in the contact above a certain carried load^15^. To mitigate this aspect, particles such as graphite or tungsten and molybdenum dichalcogenides are added to reduce friction in tribological contacts due to the very low shearing forces between their weakly bonded layers of atoms. Many studies reported an optimal concentration of 20 wt.% friction reducing constituents in self-lubricating composites, or 10–30 wt.% hard constituents for hardness and wear resistance improvements. The main disadvantage of graphite is that it is not as effective in dry conditions, as it needs water molecules to promote the shearing between its layers^16^.

An important aspect of the composites used in tribological applications is that the high temperatures/pressures generated in the contact lead to the formation of a mechanically mixed layer (MML), formed by plastic deformation of the material from both contact surfaces and subsequent oxidation of materials. The MML is found inside the wear track and is usually darker than the area outside of the contact^4,14,17^. It is composed of the initial materials in the contacting surfaces and their oxides, but its morphology is not uniform, with some areas not displaying the MML at all^4,17–20^. The removal of the MML from the wear track leads to the transition into a severe wear regime, which is undesirable. Thus, the MML has very good antiwear properties, but usually leads to a higher coefficient of friction (COF)^4,17,19,21^.

As opposed to the MMLs, which consist of plastically-deformed material and physically adsorbed wear debris particles, antiwear/extreme pressure (EP) additive fillers can react with the metal substrate to generate chemical tribofilms on the wear track. The mechanical properties of these films (e.g. lower hardness) enable a controlled wear rate of the material, while also preventing abrasion of the substrate. The reactivity of the antiwear/EP additive can be tailored to the application to avoid either the generation of thin, inefficient films or corrosion of the substrate with increased wear.

Sulphur-containing chemicals such as MoS_2_ and WS_2_ can act as both friction modifiers and antiwear additives and they can offer excellent lubricating performance for a minimal amount of corrosive wear^22^. When employed in MMCs, additives such as MoS_2_ and MoS_2_ + WS_2_ nanotubes showed very good results^2,15,23,24^. The antiwear activity is due to the ‘affinity’ of sulphur towards the fresh metal surface generated in the contact^22^. As a friction modifier, WS_2_ is superior to other solid layered structure additives because it works in both dry and humid conditions and has high temperature resistance^2,3,25–28^.

There are two types of WS_2_ particles described in the literature: 2 H (flat sheets) which have a layered structure, with W atoms confined in a trigonal prismatic coordination sphere, and IF (inorganic fullerene-like) where the layers are rounded up to form multi-layered spherical ‘onion-like’ cages.

Previous research investigating WS_2_ nanoadditives in oil showed that high pressure and temperature testing conditions (100–120 °C) promote the chemical reaction of WS_2_ nanoparticles (NPs) with the metal wear track to form a protective chemical tribofilm with favourable mechanical properties (i.e. hardness, Young’s modulus) and consequently good wear resistance^29–31^. The composition of the film and the extent of its antiwear properties are controlled by the reactivity of the additive, which is largely temperature and pressure dependent. Unreacted 2H-WS_2_ NPs and exfoliated sheets cover this tribofilm and are also responsible for a very low COF due to the weak Van der Waals forces between individual WS_2_ layers^30,31^.

IF-WS_2_ NP additives can reduce friction but are less likely to chemically interact with the metal substrate due to their structure and therefore, while the COF is reduced, the antiwear properties are inferior at these test temperatures^29,32^.

Aluminium composites containing a mixture of 3–5 wt.% 2H-WS_2_ and 5–20 wt.% SiC were shown to significantly reduce the COF and improve wear resistance in dry tests against hard materials such as steel^33,34^. 2H-WS_2_ were also found to be flexible, due to their morphology, and cover the surface of the contact well^35^.

Although WS_2_ shows significant friction reduction and wear resistance when used as particle additions in MMCs, the major difficulty lies in the preparation of the composites^3,36,37^. WS_2_ is reactive towards molten Al, and these types of MMCs were available only after the development of powder metallurgy processes, which require lower manufacturing temperatures. Controlling the temperature of the fabrication process is important in order to avoid the reaction of WS_2_ with the matrix before its operational use.

Although 2H-WS_2_ additives have been employed empirically in aluminium composites in previous studies, little is known about their mechanism of action. This study compares the performance of the 2H and IF-WS_2_ aluminium composites for the first time and investigates their mechanism of friction and wear reduction in tribological conditions similar to those encountered in engine applications. Although composites based on an aluminium alloy rather than pure aluminium would display superior properties for the mentioned applications, aluminium composites containing only 2H-WS_2_ or IF-WS_2_ particles have been used in this study to isolate the results and investigate the effect of the particles and their mechanism of action explicitly. The wear of the materials was studied with profilometry and SEM analysis and correlated to friction data from reciprocating tests. The chemical analysis of the tribofilms generated on the composite and steel ball wear tracks/scars employed Auger electron spectroscopy and X-ray photoelectron spectroscopy.

## Materials and Methods

The plate specimens used in this study were pure Al or Al MMCs reinforced with 20 wt.% 2H-WS_2_ or 20 wt.% IF-WS_2_. All specimens were consolidated using the powder metallurgy technique^38^. The process involves homogeneously mixing the base matrix and filler powders, followed by compacting at high pressure and temperature (175 MPa and 550 °C). Previous studies on WS_2_ particles have shown that they are stable to very high temperatures and even display a self-cleaning process by impurity atom removal in temperature ranges of 450 °C^39^. The reaction of WS_2_ with the metal substrate has been indicated to require sliding conditions in addition to high local contact temperatures between asperities^30,31^, whereas a reaction initiated only due to temperature was described in the literature to occur above 700 °C for MoS_2_, which has a similar structure, and at a possibly higher temperature for WS_2_^16^.

2H-WS_2_ particles were purchased from Sigma Aldrich (average size 2 μm, 99%). IF-WS_2_ NPs were produced by a continuous reaction of WO_3_ (average size 50 nm, Sigma Aldrich) with H_2_S gas (BOC, UK) in Ar (BOC, UK) atmosphere at 800 °C, in a newly designed rotary furnace, as previously described^40^.

The Vickers hardness of the materials was measured at 10 kg load for 15 s loading time (Vickers HTM 4133). The elastic modulus was determined using a Shimadzu DUH-211 dynamic ultra-micro hardness tester, with a normal load of 50 mN (the indentation depth is about 2 µm), while Poisson’s ratio was set at 0.35.

Friction and wear properties were determined using a ball-on-plate type test rig at 25 and 100 °C. A 6.35 mm diameter steel ball is held in a chuck and loaded against a flat face of the composite immersed in oil. The material used for the balls is JIS SUJ2 steel, equivalent to AISI 52100 steel. The normal load (10 N) generated a contact pressure of 0.87 GPa on the pure Al and 0.72 GPa on the WS_2_ composites due to the different elastic modulus values of the specimens. The tests were carried out under the conditions listed in Table 1.

**Table 1:** Reciprocating test conditions.

Stroke length	6 mm
Stroke frequency	1.66 Hz (100/min)
Load	10 N
Mean Hertz pressure	0.87/0.72 GPa
Entrainment speed	20 mm/s
Test duration	60 min
Temperature	25 °C and 100 °C
Ball properties	JIS SUJ2 steel
Disc properties	IF/2H-WS_2_ Al MMC

The tests were conducted with a synthetic oil (PAO), which has a density of 0.826 g/cm^3^ and a viscosity of 28.8 cSt at 40 °C and 5.6 cSt at 100 °C (~54.6 cSt at 25 °C). Polyalphaolefins have low polarity and thus low affinity for metal surfaces, which avoids any competition between the WS_2_ particles and the oil molecules for the metal substrate, as discussed in the literature^30^.

The surfaces of the specimens were first ground with silicon carbide paper followed by buff polishing with 3-μm diamond slurry. A single ball and disc was used for each test and they were ultrasonically cleaned with toluene and isopropanol prior to use.

Optical profilometry (Bruker ContourGT) was used to study the depth and morphology of the wear tracks on the disc specimens after each test. The equipment generates a 3D surface profile of the wear track that can be analysed with the supplied software. Multiple surface profiles were measured for each image and the average depth values were calculated.

XPS analysis was performed on/off the wear track of the aluminium composites at the end of the tests to study the generation of the tribofilms, their elemental composition and chemical state of the elements. The analysis was carried out at room temperature and employed a Thermo Scientific K-Alpha spectrometer (East Grinstead, UK) with a 1486.6 eV microfocused monochromatic Al (Kα) X-ray source. The spot size was 200 × 400 μm^2^ (ellipse shape). The pass energy was 200 eV for the wide (survey) spectra and 40 eV for the high resolution regions (narrow spectra). The instrument features an argon gun, which was used to clean the samples by sputtering. The raster size for the sputtering was 1 × 2 mm^2^, using the Ar gas cluster ion beam (GCIB) mode at 6 keV with 1000 atoms for 60 s and an estimated 2 nm of material was removed from the surface.

An Auger electron spectrometer (JEOL JAMP 9500 F) with a 10 keV electron beam was used to investigate the elemental distribution across the wear scar on the ball specimens. Ion sputtering was performed before the analysis in order to remove the contaminated layer on the surface (an estimated depth of ~2 nm was removed). The depth profiles were obtained by 30 s sputtering cycles alternating with Auger elemental analysis. Prior to the analysis, the surface of the wear tracks was cleaned with solvents in order to remove the oxidized oil. The areas analysed with Auger were visualized by SEM imaging.

## Results and Discussion

**Figure 1:**
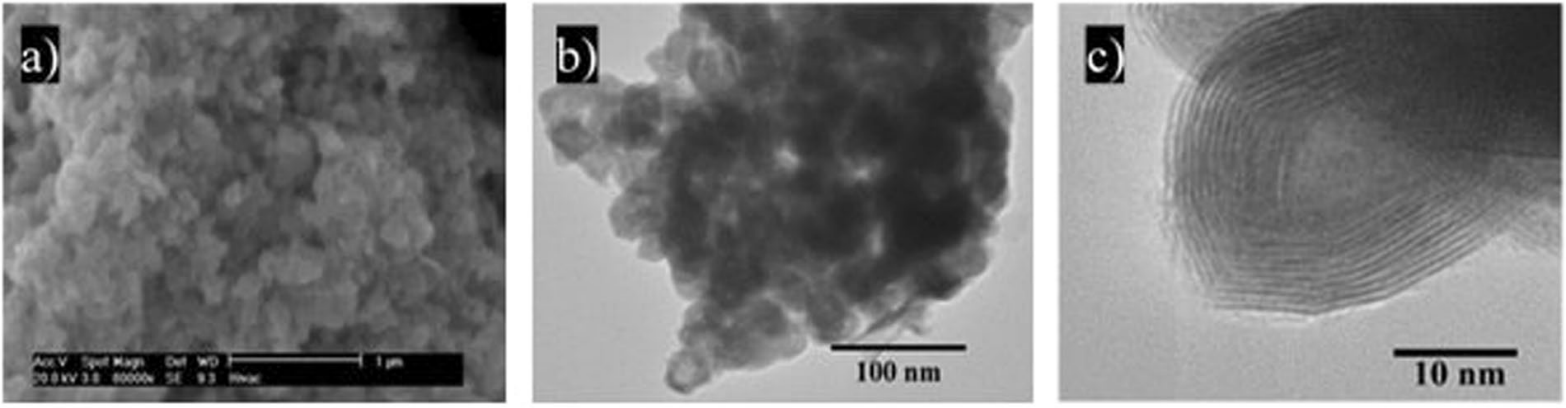
SEM (**a**) and TEM (**b**,**c**) images of IF-WS_2_ nanoparticles.

**Figure 2:**
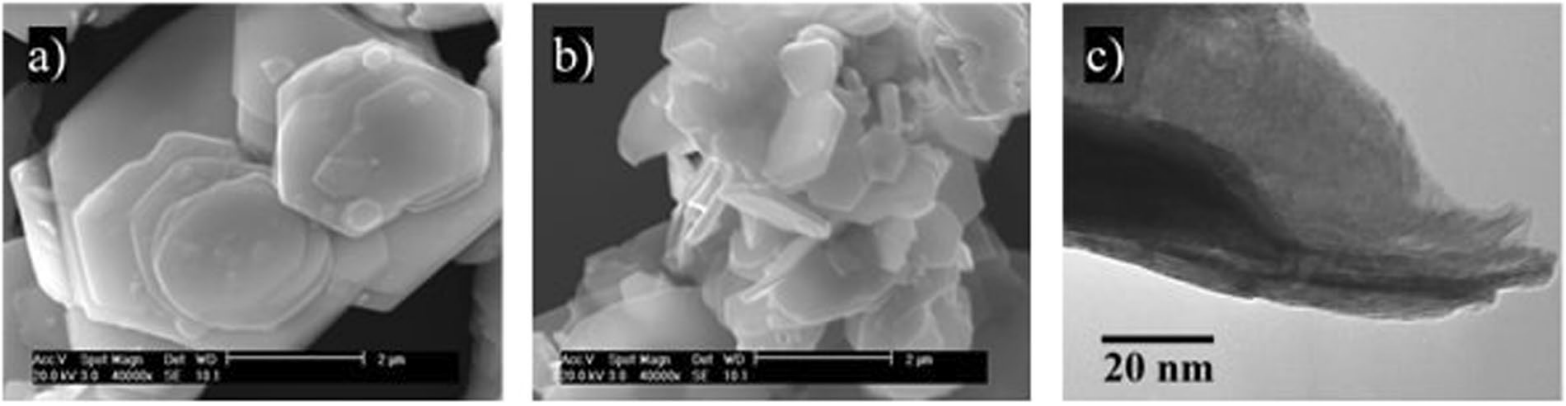
SEM (**a**,**b**) and TEM (**c**) images of commercial 2H-WS_2_ particles.

SEM and TEM images of the IF-WS_2_ and 2H-WS_2_  particles, shown in Figs 1 and 2, reveal the hexagonal shape of the 2H-WS_2_ particles (due to the trigonal prismatic geometry of the WS_2_ sheets), which had sizes of between 1–5 µm, while the synthetized IF-WS_2_ NPs have a narrow size distribution with an average size of approximately 50 nm. The particles appear as aggregates in TEM images due to the preparation process of the TEM grids, which requires the particles to be dispersed in solvent.

**Figure 3:**
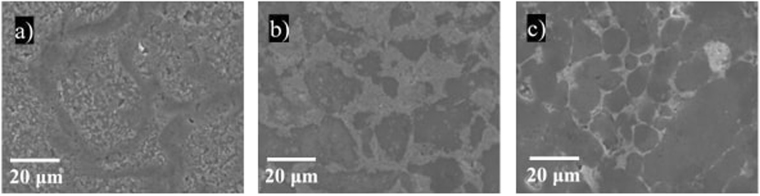
SEM images showing the structure of: (**a**) Aluminium, (**b**) Aluminium with 20 wt.% IF-WS_2_ composite and (**c**) Aluminium with 20 wt.% 2H-WS_2_ composite.

SEM images of the microstructure of the composites show a large grain size in the range of tens of micrometers (Fig. 3). Previous research has shown that nanograin effects (i.e. when the grain size is in the nanometer scale) can generate superior mechanical properties due to the higher density of the material or the reduced probability of defects in the structure. The micro grain size of the composites investigated in this study assures that the mechanical properties of the composites are not influenced by nanograin size effects^41^.

The hardness and elastic modulus values essential for tribological studies, for the three composites, are presented in Table 2. The presence of the particle additions in the Al matrix results in a small increase in hardness and a decrease in the elastic modulus. The similar mechanical properties of the two composites suggests that the size of the particles does not have a large effect on the bulk material properties of the composite.

**Table 2:** Mechanical properties of the materials.

	Hardness (HV)	Elastic modulus (GPa)
Al	34	71.7
Al-IF-WS_2_	39	49.6
Al-2H-WS_2_	45	50.1

The particle content may not be optimum for the mechanical properties of the composites, but in this application the importance of the tribological performance of the composite is paramount, as the failure mechanism will be directly related to the efficiency of the composite to resist wear processes.

Composites based on an aluminium alloy rather than pure Al would display superior tribological properties, but Al composites containing only WS_2_ have been employed in this study. The presence of other alloying elements and thus precipitates in the aluminium matrix can interfere with the mechanism of action, i.e. the high temperature-induced chemical reactions between WS_2_ and the Al matrix that result in the generation of a chemical tribofilm. The performance of the tribofilm can be greatly influenced by the presence of other particles present in the matrix.

**Figure 4:**
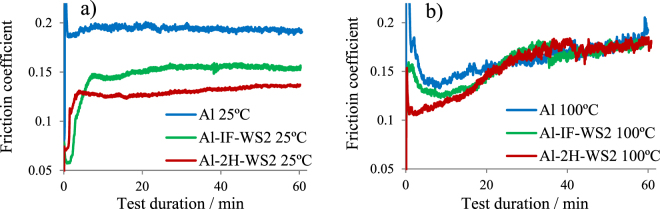
Average friction coefficient of the stroke length during the tests performed at 25 °C (**a**) and 100 °C (**b**).

Figure 4(a) and (b) show the average COF during each sliding test plotted against time. At 25 °C (Fig. 4(a)) both IF-WS_2_ and 2H-WS_2_ decrease the COF of the Al composite, with 2H-WS_2_ showing the largest friction reduction (30%). At 100 °C (Fig. 4(b)), the WS_2_ particles have a beneficial effect on friction in the first 20 minutes of the test, after which the COF increases to similar values as those displayed by the pure Al disc specimens. The friction reduction effect of 2H-WS_2_ is more prominent, which can result in a superior load-carrying capacity, due to the presence of sulphur in the tribofilm^22^, similar to other findings in the literature for these types of loads^42^. The increase in the COF during the test for both, pure Al and the two Al composites is the result of different factors. For the pure Al sample this is ascribed to the gradual increase of the contact area, while for the WS_2_-containing composites it could be due to the larger transfer of material on the ball and the characteristics of the chemical tribofilm generated on the composite, i.e. high roughness and hardness linked to high friction^19,30,31^. These processes contribute to the complex interactions between the two contacting surfaces. The increased friction is a compromise for providing antiwear performance through the chemical tribofilm. By this action, the corrosion of the substrate is controlled and the occurrence of sudden failure due to adhesion is prevented.

To study the effect of the WS_2_ additive particles on the antiwear performance of the Al composites, the wear tracks were investigated using optical profilometry and SEM at the end of the tribological tests. The former enabled the measurement of the widths and depths of the wear tracks, while the latter was used for the detailed visualisation of their structure.

**Figure 5:**
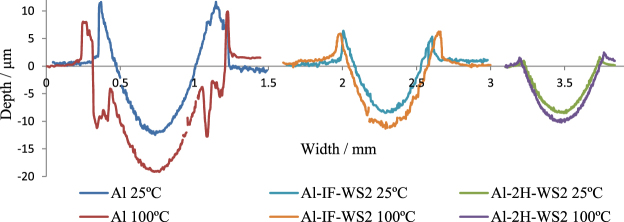
Wear track profiles on the composite samples.

Figure 5 shows the wear track of the Al composites. The pure Al specimens displayed the largest amount of wear at both 25 and 100 °C. The shape of the profiles is irregular and there is also a large amount of material piled at the sides of the wear track due to plastic deformation. The wear of the WS_2_ composites was significantly reduced and the profile of the tracks was smoother, particularly for the 2H-WS_2_ composite. This situation is commonly encountered with antiwear additives used for lubrication of steel counterparts and is due to their chemical reaction with the metal substrate, with the generation of low-strength sacrificial films that wear in a controlled manner. The wear track width (L) and the wear coefficient (K), calculated as the total wear volume divided by the load and sliding distance for the three composites tested at 25 and 100 °C are presented in Table 3.

**Table 3:** Wear track width and wear coefficient for the three composites.

	25 °C		100 °C	
	L (µm)	K (m^2^/N)	L (µm)	K (m^2^/N)
Al	750	4.6 × 10^−8^	900	8.6 × 10^−8^
Al-IF-WS_2_	650	2.7 × 10^−8^	650	3.6 × 10^−8^
Al-2H-WS_2_	550	2.3 × 10^−8^	550	2.8 × 10^−8^

Similar to the results obtained with the WS_2_ lubricant additive, in the case of WS_2_ composites, the smoother wear track profiles and the small amount of material piled up at the sides indicate that the 2H-WS_2_ composite samples exhibited superior antiwear properties compared to the IF-WS_2_ composites in the conditions employed in this study. The reason could be their faster reaction kinetics and the generation of tribofilms with a different chemical composition.

**Figure 6:**
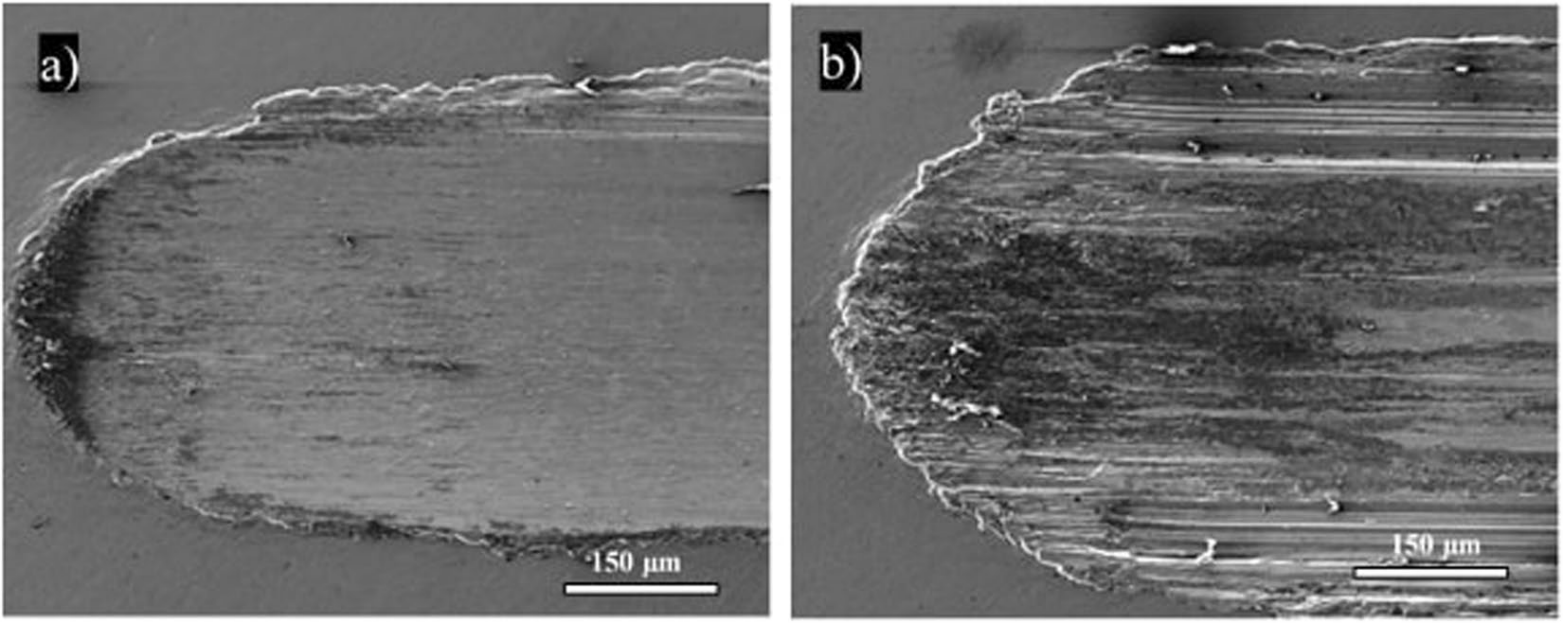
SEM images of the wear tracks on the pure Al specimen tested at 25 °C (**a**) and 100 °C (**b**).

**Figure 7:**
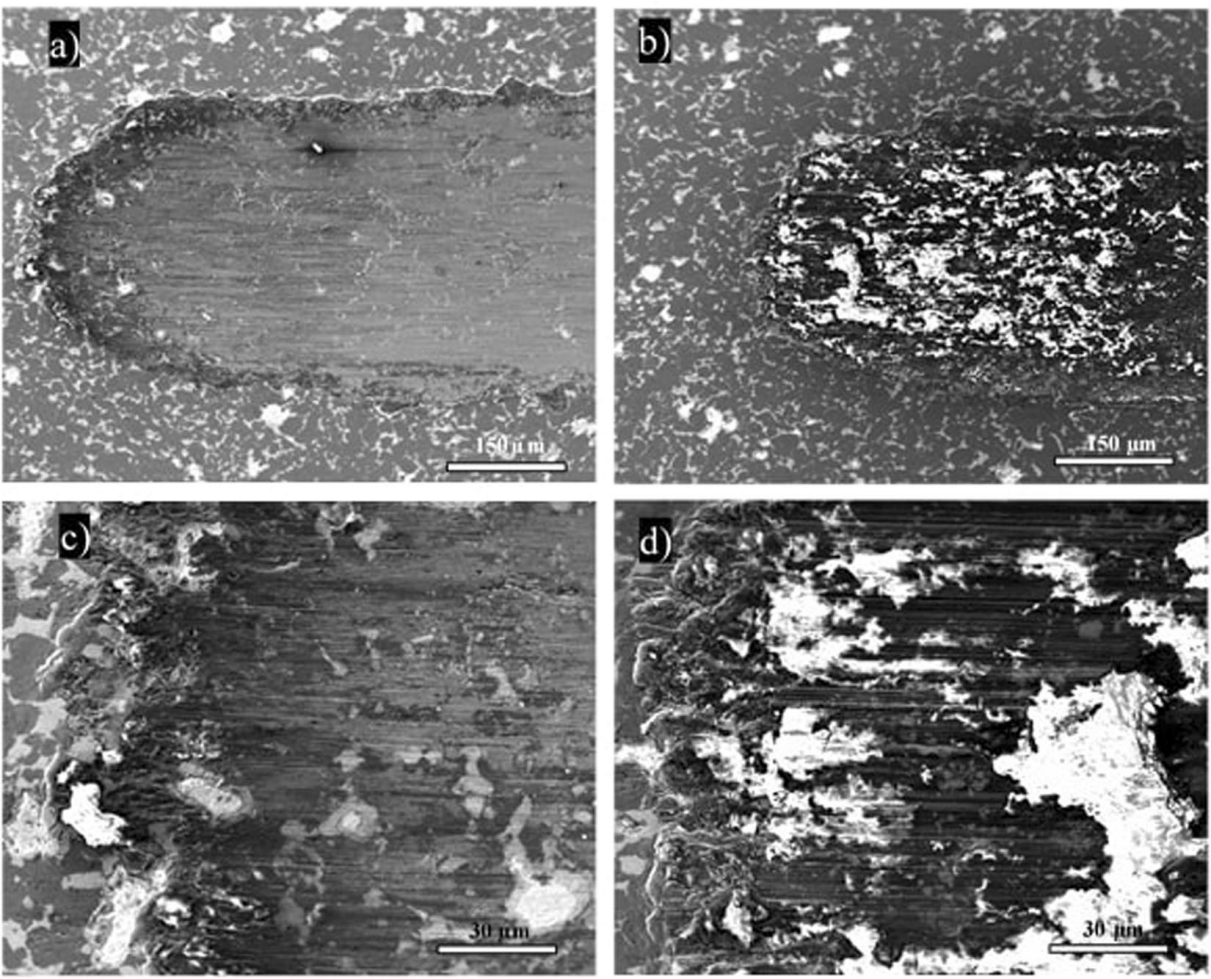
SEM images of the wear tracks on the IF-WS_2_ composite tested at 25°C (**a**,**c**) and 100 °C (**b**,**d**).

**Figure 8:**
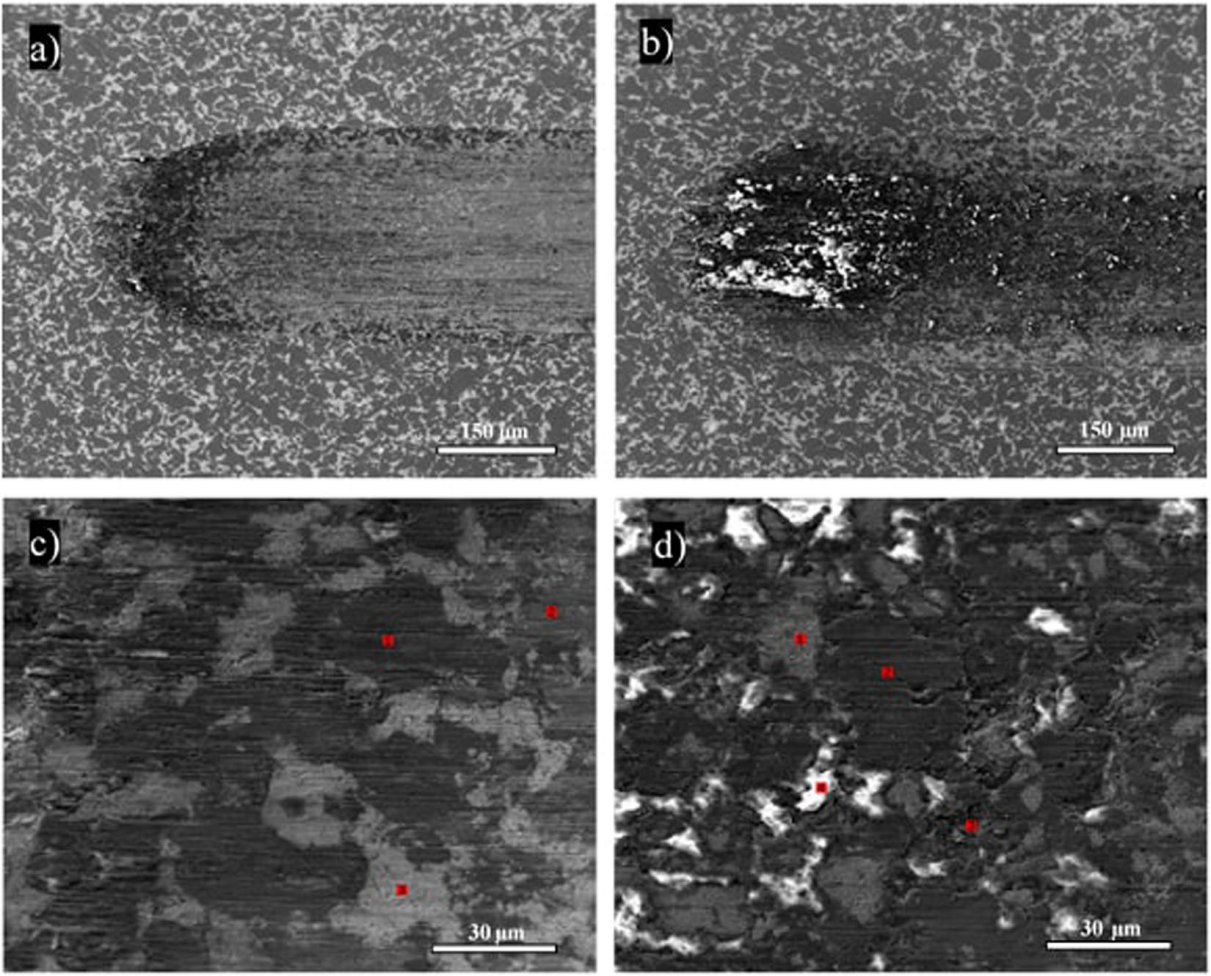
SEM images of the wear tracks on the 2H-WS_2_ composite tested at 25 °C (**a**,**c**) and 100 °C (**b**,**d**).

Figures 6,7 and 8 show SEM images of the wear tracks of the three composites at the end of the test. Similarly to the profilometry measurements, the SEM images show that the width of the composite wear tracks follows the trend: Al > IF-WS_2_ > 2H-WS_2_.

The wear tracks on the pure Al specimens (Fig. 6) appear larger and rougher than the WS_2_ additized composites. These differences are more prominent at 100 °C (Fig. 6(b)).

The wear tracks generated on the IF-WS_2_ tracks at the two testing temperatures are considerably different (Fig. 7). At 100 °C the track is covered by a MML consisting of a dark material with bright white spots, a possible indication of a chemical reaction.

The morphology of the 2H-WS_2_ composites (Fig. 8) has a similar appearance. The tracks generated at 100 °C are covered by a MML with dark and bright white spots but the extent of these dark areas is larger and more prominent. This suggests that the tribofilm may have a different chemical composition. Previous work has shown than 2H-WS_2_ NPs react with a steel wear track and generate white/dark areas during rubbing^43^. Auger electron spectroscopy analysis showed that the white areas were rich in iron sulphides, while the dark areas contain large amounts of tungsten trioxide (WO_3_).

Both SEM images and profilometry of the wear tracks showed that the antiwear properties of the composites were improved with the inclusion of WS_2_ particles in the matrix. The smoother and narrower wear tracks of the WS_2_ composites imply a possible reaction of WS_2_ with the aluminium matrix and the formation of a chemical tribofilm which improves tribological properties. To investigate this hypothesis, chemical analysis (XPS) was performed on the composite wear tracks.

**Figure 9:**
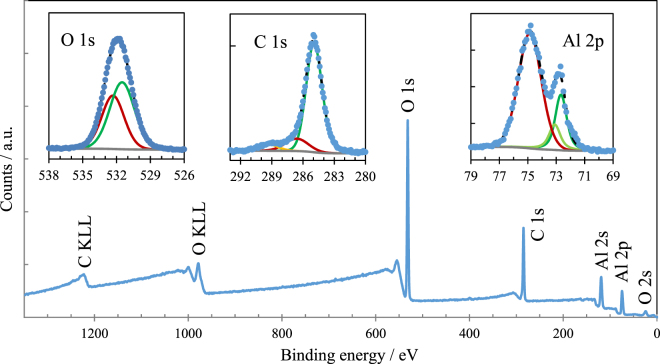
XPS spectra recorded inside the track on the pure Al sample.

Figure 9 shows the wide XPS spectrum recorded inside the wear tracks on the Al specimens tested at 25 °C. The chemical analysis reveals the presence of aluminum, carbon (from decomposed oil molecules) and oxygen (as Al oxide and from decomposed oil). The colours of the signal traces in the figure show the contributions of the chemical species present. Similar colours show the orbital contributions of the same chemical species (e.g. 2p 1/2 and 2p 3/2).

The narrow spectra for O, C and Al are presented in the insets in Fig. 9 and the position of the fitted signals is shown in Table 4. The signals recorded for O 1s were assigned to metal oxides (531.3 eV) and a large contribution from C-O bonds from oxidized oil molecules (532.1 eV). The signals recorded for the C contamination products are due to C-C bonds (284.8 eV), C-O bonds (286.4 eV) and C=O bonds (289.1 eV). Aluminium is mostly found as Al_2_O_3_ (74.8 eV) and also as Al° (72.6 eV). The analysis performed at 100 °C generated similar results.

**Table 4:** XPS signals recorded on the wear track of the pure Al sample.

	Al		C (contamination)			O	
	Al°	Al^3+^ (Al_2_O_3_)	C in C-C	C in C-O	C in C=O	O in Al_2_O_3_	O as contamination
Energy (eV)	72.6	74.8	284.8	286.4	289.1	531.3	532.1

For the IF-WS_2_ and 2H-WS_2_ composites, the XPS measurements were performed both inside and outside the wear track.

The analysis performed outside the wear track for both composites found only Al, O and C. This indicates that the chemical tribofilm is only generated on the wear track and that apart from temperature, it requires high pressure and possibly shear (non-zero slide-roll ratio) to form.

**Figure 10:**
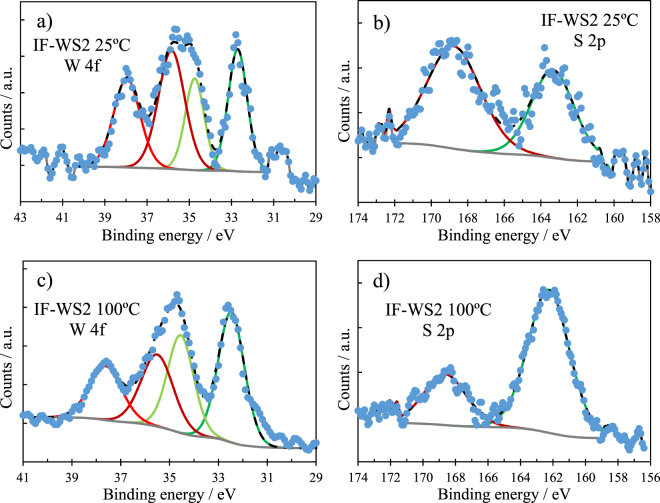
XPS spectra recorded inside the wear track of Al-IF-WS_2_ composites tested at 25 °C and 100 °C; (**a**,**c**) W 4f spectra; (**b**,**d**) S 2p spectra.

The XPS analysis inside the wear tracks is presented in Fig. 10 (for IF-WS_2_) and 11 (for 2H-WS_2_) and Table 5. For tungsten, W^0^ (elemental W) is expected at ~31 eV, W^4+^ (in WS_2_) at ~33 eV and W^6+^ (in WO_3_) at ~35.5 eV^32,44–46^.

**Table 5:** XPS signals recorded on the wear track of IF-WS_2_ and 2H-WS_2_ composites.

Energy (eV)	W			S	
	W^0^	W^4+^	W^6+^	S^2−^ (sulphide)	S^6+^ (sulphate)
IF-WS_2_	—	32.5	35.6	162.2	168.6
2H-WS_2_	30.9	33.1	35.6	162.5	169.1

Figure 10 shows the narrow XPS spectra for W 4f and S 2p on the wear track formed on the IF-WS_2_ composite at 25 and 100 °C. At both temperatures, the W 4f signal appears as a doublet with an energy gap of ~2.15 eV.

On the track generated at 25 °C, tungsten (Fig. 10(a)) was mainly found as W^6+^ (in WO_3_) at 35.6 eV and W^4+^ (WS_2_) at 32.5 eV. The S 2p signal (Fig. 10(b)) shows sulphides (162.2 eV) and sulphur oxides/sulphates (168.6 eV). W^0^ was not detected. There is a strong signal for W^4+^ indicating that the wear track on the IF-WS_2_ composite contains WS_2_, as NPs or exfoliated sheets.

The narrow spectra for W 4f and S 2p measured on the wear track generated at 100 °C are similar to those measured at 25 °C, suggesting that this temperature increase does not influence the reactivity of IF-WS_2_ NPs.

**Figure 11:**
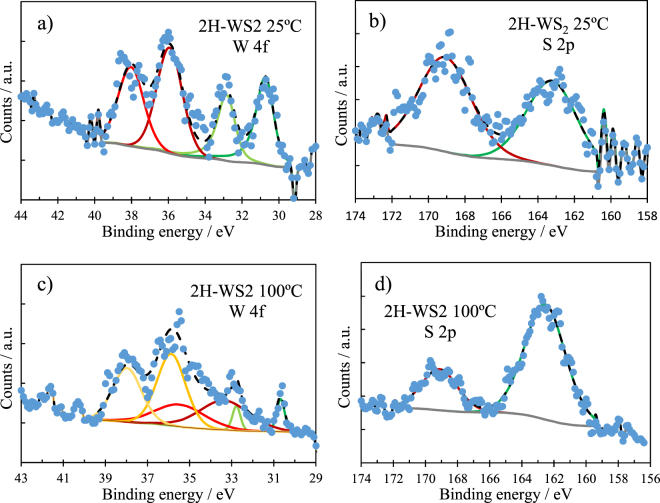
XPS spectra recorded inside the wear track of Al-2H-WS_2_ composites tested at 25 °C and 100 °C; (**a**,**c**) W 4f spectra; (**b**,**d**) S 2p spectra.

Figure 11 shows the narrow XPS spectra for W 4f and S 2p recorded on the 2H-WS_2_ composite wear tracks. The wear track tested at 25 °C (Fig. 11(a)) shows signals for tungsten, corresponding to W^6+^ (WO_3_) at 35.6 eV and W^0^ (elemental tungsten) at 31.2 eV. The presence of tungsten as WO_3_ and W^0^ in the tribofilm can only result from a chemical reaction between the 2H-WS_2_ particles and the metal substrate (the Al matrix or the steel ball counterface). However, no iron signal was detected on the wear track, implying that 2H-WS_2_ chemically reacted during rubbing with the Al matrix.

The two S 2p peaks in Fig. 11(b) were attributed to sulphur oxides/sulphates (169.1 eV) and sulphide (162.5 eV). The lack of a visible W^4+^ signal corresponding to WS_2_ in Fig. 11(a) implies that this must be aluminium sulphide generated by the chemical reaction of the particles with the substrate.

The narrow spectra of W 4f and S 2p at 100 °C are similar to those found at 25 °C with the only difference of an additional signal observed for W^4+^ (WS_2_) at 33.1 eV on the wear track formed at 100 °C (Fig. 11(c)). The position of the measured signals are shown in Table 5.

The XPS results indicate that the tribofilm generated on the wear track of the 2H-WS_2_ composite results from a chemical reaction between 2H-WS_2_ and the aluminium matrix. The presence of tungsten compounds such as W^0^ and WO_3_ has been previously shown^30,31^ to improve the antiwear properties of the tribofilm, which can lead to shallower and smoother wear tracks on the Al composites, as seen in Fig. 5.

The relative atomic concentration of S and W in the tribofilms formed on the WS_2_ composites can assess the reactivity of the WS_2_ particles with the aluminium matrix in the tribological contact. We have previously shown^30,31^ than the reaction of WS_2_ NPs used as additive in oil lubricants with the bearing steel wear track leads to the formation of a 100+ nm layered tribofilm, which contains large amounts of S compounds in the upper part and a high concentration of W compounds in the deeper layers of the film. Therefore, a non-stoichiometric distribution of the two elements in the tribofilm indicate a chemical reaction.

Table 6 shows the S:W ratio on the wear tracks as measured with XPS in the upper part of the tribofilm. The film on the IF-WS_2_ composite displays a stoichiometric S:W ratio of approximately 2:1 at both testing temperatures (25 and 100 °C), which indicates that the chemical reaction is impeded. By contrast, the film on the 2H-WS_2_ composite contains a significantly larger amount of sulphur at both testing temperatures, 25 °C (~4:1) and 100 °C (~5:1), indicating the formation of sulphur-containing compounds at the top of the tribofilm due to a chemical reaction between 2H-WS_2_ and the Al matrix.

**Table 6:** S:W ratio on the wear tracks of the 2H and IF-WS_2_ composites.

	2H-WS_2_	IF-WS_2_
25 °C	3.95	2.07
100 °C	5.06	2.06

**Figure 12:**
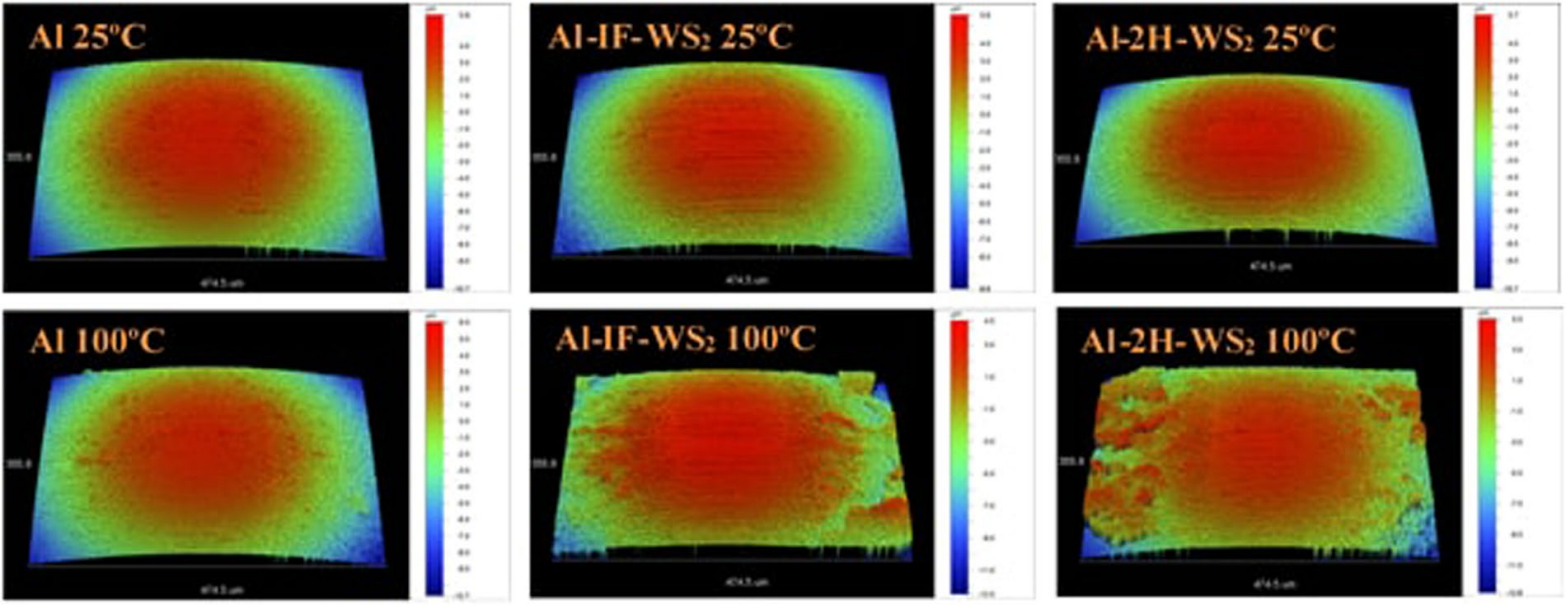
Profilometry images of the steel ball wear scars.

Optical profilometry images of the steel balls rubbed against the pure Al and Al composites display the wear scar on an area of 355 × 475 μm^2^ and depth of about 15 μm (Fig. 12). At 100 °C, a significantly larger amount of wear scar material was observed on the balls rubbed against the 2H- and IF-WS_2_ composites, as compared to the pure Al specimen. The characteristics and composition of these tribofilms are important for understanding the mechanism of action of the composites and for this reason, the steel balls rubbed against the WS_2_ composites at 100 °C were additionally imaged by SEM, while the chemical composition of the tribofilms was investigated with Auger spectroscopy.

**Figure 13:**
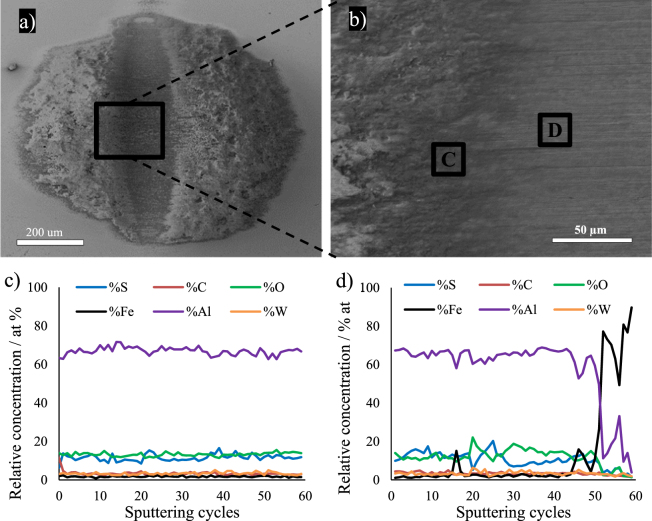
(**a**,**b**) SEM images of the wear scar on the ball tested against the IF-WS_2_ composite at 100 °C; (**c**,**d**) Auger ion sputtering depth profile of the chemical composition in points C and D of the tribofilm.

**Figure 14:**
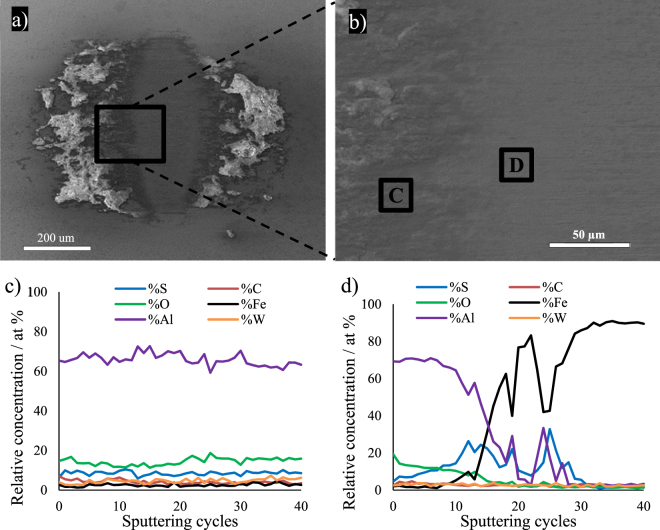
(**a**,**b**) SEM images of the wear scar on the ball tested against the 2H-WS_2_ composite at 100 °C; (**c**,**d**) Auger ion sputtering depth profile of the chemical composition in points C and D of the tribofilm.

SEM images of the wear scars on balls rubbed against the IF-WS_2_ (Fig. 13(a)) and 2H-WS_2_ (Fig. 14(a)) composites show an elliptical shaped central area with a smooth surface. The tribofilm material is abundant at both sides of the central area. Figures 13(b) and 14(b) show the left boundary of the central area. The wear scar is larger for the ball rubbed against the IF-WS_2_ than the 2H-WS_2_ composite (Table 7), in agreement with the wider wear track measured on the IF-WS_2_ composite (Table 3). Furthermore, the SEM images confirm the optical profilometry observation that a larger amount of wear scar material is detected on the ball rubbed against the IF-WS_2_ than the 2H-WS_2_ composite.

**Table 7:** Physical properties of the wear scars on the ball specimens tested at 100 °C.

	Central wear scar (µm)	Outer wear scar (µm)	Surface roughness (nm)
IF-WS_2_	150 × 630	840 × 630	115
2H-WS_2_	230 × 520	750 × 520	120

Figure 13(c) and (d) show the elemental composition with depth of the tribofilm formed on the steel ball rubbed against the IF-WS_2_ composite measured using Auger spectrometry in the central area boundary at points C and D (Fig. 13b)). In point C, outside the central wear scar (Fig. 13(c)), the tribofilm shows a constant composition of Al, S and O, indicating the presence of a mixture of aluminium oxide (Al_2_O_3_) and aluminium sulphide (Al_2_S_3_). Other elements (C, Fe and W) are only detectable as trace amounts. A similar composition is found in point D, inside the central area, but the film was thinner and the steel substrate was reached after ~50 sputtering cycles (Fig. 13(d)).

The composition of the tribofilm on the ball rubbed against the 2H-WS_2_ composite is shown in Fig. 14(c) and (d). At point C, outside the central area of the wear scar, a mixture of Al_2_O_3_ and Al_2_S_3_ similar to that formed by the IF-WS_2_ composite on the ball is found. However, inside the scar (point D), a larger amount of metal sulphides (most probably Al_2_S_3_, as the presence of tungsten is negligible) is detected at the interface with the steel substrate. The ion sputtering analysis indicated that the steel substrate was reached after 20–30 sputtering cycles.

Therefore, the steel substrate at point D of the central area was reached after 20–30 sputtering cycles in the case of 2H-WS_2_ compared to 50 cycles for IF-WS_2_. These results are in agreement with the profilometry and SEM observations that the material transferred on the ball for the 2H-WS_2_ composite is visibly thinner than that for the IF-WS_2_ composite.

The tribofilms contain chemical reaction products and wear debris generated during rubbing and therefore have a complex amorphous composition. Due to the uneven distribution of material and the varying sputtering rates of the chemical species present in the tribofilm, it is difficult to estimate the amount of material removed during the sputtering process and the depth profiles can only be presented as a function of sputtering cycles. However, other published studies that investigated the antiwear properties of WS_2_ found the thickness of the tribofilms in the range of 100+ nm^30,31^.

The composition of the tribofilms support the hypothesis that they were transferred on the steel balls from the aluminium composites during rubbing. It also confirms the generation of chemical tribofilms on the composite wear track during rubbing through the reaction of WS_2_ particles with the Al matrix. Sulphur-containing antiwear additives usually generate tribofilms rich in sulphides on metal substrates due to the high reactivity of the sulphur atom towards metal interfaces and these tribofilms have a superior antiwear/extreme pressure performance^22^.

The difference in the size of the wear scar and the thicknesses and composition of the tribofilms generated in the contact can be explained by the distinct mechanism of action of the WS_2_ particles. At high temperature, the 2H-WS_2_ composite generates a stronger chemical tribofilm which controls the wear rate more efficiently. In the case of IF-WS_2_ the chemical reaction is inhibited and the mechanism of action is predominantly physical, as in the case of conventional solid friction modifiers. Therefore, these tribofilms have a weaker interaction with the substrate and result in more wear debris generation, significantly larger wear tracks and more transferred material as observed on the steel balls after the tests.

In the test conditions employed in this study (relevant for engine applications) the 2H-WS_2_ particles showed superior performance. However, applications that require higher temperature/pressure conditions could promote the chemical reaction of the less reactive IF-WS_2_ particles with the Al matrix and formation of a chemical tribofilm similar to the 2H-WS_2_ particles. Further work is required to investigate this.

## Conclusions

This study has investigated the mechanism of action of Al-MMCs containing 2H-WS_2_ microparticles and IF-WS_2_ nanoparticles in lubricated contacts against a steel ball in high pressure (0.7 GPa) reciprocating contacts.

The results show that Al MMCs containing WS_2_ have improved friction and wear properties in sliding contacts compared to pure Al. Under the conditions employed in this study, i.e. temperatures of 25 and 100 °C and a contact pressure of 0.72 GPa, the composites containing IF-WS_2_ or 2H-WS_2_ particle additions displayed a COF reduced by ~20% and ~30% respectively, along with narrower, shallower and smoother wear tracks than the pure Al specimens.

The ability of the composites to exhibit reduced friction can be explained by the exfoliation of the layer-structured WS_2_ particles under shear, consistent with published results obtained using WS_2_ nanoadditized lubricants.

The antiwear performance is attributed to the ability of WS_2_ particles to react with the Al matrix and generate a chemical tribofilm on the wear track. The XPS analysis of the tribofilms revealed that in the conditions tested, 2H-WS_2_ particles react more readily than IF-WS_2_ with the aluminium matrix to generate the tribofilm. These findings explain the superior antiwear behaviour of the 2H-WS_2_ composites and are in agreement with those obtained when steel contacts were lubricated with 1 wt.% WS_2_ (2H- or IF-WS_2_) additized oils under similar testing conditions.

During rubbing, the chemical tribofilm generated on the WS_2_ composites wear track was transferred to the steel balls wear scar. The smaller wear scar and thinner transferred film obtained for the 2H-WS_2_ composite is explained by the chemical nature of the 2H-WS_2_ generated antiwear tribofilm, which is strongly attached to the composite surface and therefore provides a lower wear rate.

The use of WS_2_ in Al alloys can significantly improve their resistance to seizure and galling and lead to lighter, more efficient and greener tribological components. Due to its mechanism of action and economical aspects, 2H-WS_2_ particles are suitable additions to Al composites operating in the tribological conditions investigated in this study.
